# A novel inhibitor of fatty acid synthase shows activity against HER2+ breast cancer xenografts and is active in anti-HER2 drug-resistant cell lines

**DOI:** 10.1186/bcr3077

**Published:** 2011-12-16

**Authors:** Teresa Puig, Helena Aguilar, Sílvia Cufí, Glòria Oliveras, Carlos Turrado, Sílvia Ortega-Gutiérrez, Bellinda Benhamú, María Luz López-Rodríguez, Ander Urruticoechea, Ramon Colomer

**Affiliations:** 1 Institut d'Investigació Biomèdica de Girona, E-17071 Girona, Spain; 2 Facultat de Medicina, Universitat de Girona, E-17001 Girona, Spain; 3 Institut Català d'Oncologia - Institut d'Investigació Biomèdica de Bellvitge, E-08907 Barcelona, Spain; 4 Química Orgánica I, Facultad de Ciencias Químicas, Universidad Complutense, E-28040 Madrid, Spain; 5 Centro Oncológico MD Anderson España, E-28033 Madrid, Spain

## Abstract

**Introduction:**

Inhibiting the enzyme Fatty Acid Synthase (FASN) leads to apoptosis of breast carcinoma cells, and this is linked to human epidermal growth factor receptor 2 (HER2) signaling pathways in models of simultaneous expression of FASN and HER2.

**Methods:**

In a xenograft model of breast carcinoma cells that are FASN+ and HER2+, we have characterised the anticancer activity and the toxicity profile of G28UCM, the lead compound of a novel family of synthetic FASN inhibitors. *In vitro*, we analysed the cellular and molecular interactions of combining G28UCM with anti-HER drugs. Finally, we tested the cytotoxic ability of G28UCM on breast cancer cells resistant to trastuzumab or lapatinib, that we developed in our laboratory.

**Results:**

*In vivo*, G28UCM reduced the size of 5 out of 14 established xenografts. In the responding tumours, we observed inhibition of FASN activity, cleavage of poly-ADPribose polymerase (PARP) and a decrease of p-HER2, p- protein kinase B (AKT) and p-ERK1/2, which were not observed in the nonresponding tumours. In the G28UCM-treated animals, no significant toxicities occurred, and weight loss was not observed. *In vitro*, G28UCM showed marked synergistic interactions with trastuzumab, lapatinib, erlotinib or gefitinib (but not with cetuximab), which correlated with increases in apoptosis and with decreases in the activation of HER2, extracellular signal-regulated kinase (ERK)1/2 and AKT. In trastuzumab-resistant and in lapatinib-resistant breast cancer cells, in which trastuzumab and lapatinib were not effective, G28UCM retained the anticancer activity observed in the parental cells.

**Conclusions:**

G28UCM inhibits fatty acid synthase (FASN) activity and the growth of breast carcinoma xenografts *in vivo*, and is active in cells with acquired resistance to anti-HER2 drugs, which make it a candidate for further pre-clinical development.

## Introduction

Fatty acid synthase (FASN) is a multifunctional enzyme that is essential for the endogenous synthesis of long-chain fatty acids from its precursors acetyl-CoA and malonil-CoA [1]. Blocking FASN activity causes cytotoxicity in human cancer cells overexpressing FASN [2-13]. The proposed oncogenic properties of FASN seem to be the result of an increased activation of HER2 and its downstream related phosphoinositide-3 kinase/protein kinase B (PI3K/AKT) and mitogen-activated protein kinase/extracellular signal-regulated kinase (MAPK/ERK1/2) signalling cascades or to the mammalian target of rapamycin protein (mTOR) signaling pathway [4,5,8,13-20]. FASN can also inhibit the intrinsic pathway of apoptosis [21] and has been recently proposed as a direct target of p53 family members, including p63 and p73 [22]. FASN inhibition may also disrupt the membrane lipid rafts that anchor HER2 [23]. In the past, FASN inhibitors with antitumour activity have been limited by either cross-activation of β-oxidation, which produces *in vivo *anorexia and body weight loss [9,24-28], or low potency [29,30].

The molecular mechanisms of resistance to anti-HER2 therapies in breast carcinomas have been reviewed recently [31,32]. These include loss of PTEN [33], predominance of the p95^HER2 ^expression [34], mTOR/PI3K/AKT hyperactivation [35], IGF-IR overexpression [36], and *in vivo *conversion of HER2+ to HER2- carcinoma after neoadjuvant trastuzumab [37]. The limited experimental evidence available shows that, in cancer cells, a cross-regulation between FASN and HER2 exists [3,5], and also that pharmacological blockade of FASN with C75 can overcome acquired resistance to trastuzumab [38].

We have recently described a novel family of anti-FASN compounds that exhibit *in vitro *anticancer activity, which do not exhibit cross-activation of β-oxidation, and do not induce weight loss in animals [13]. In the current study, we have characterised molecularly the *in vivo *anticancer activity of G28UCM in a model of FASN+/HER2+ breast carcinoma. In addition, we have evaluated the pharmacological interaction of G28UCM with anti-HER drugs, such as trastuzumab, lapatinib, erlotinib, gefitinib or cetuximab, at the cellular and molecular levels. Finally, we report the effect of G28UCM on breast cancer cells resistant to trastuzumab or lapatinib. Our data support the study of G28UCM as a potential therapeutic agent, either alone or in combination, against *in vivo *HER2+ tumours that have progressed on trastuzumab and lapatinib.

## Materials and methods

### Chemicals, reagents and antibodies

Erlotinib (Tarceva^®^), gefitinib (Iressa^®^) and lapatinib (Tyverb^®^) were provided by Roche (Roche, London, UK), AstraZeneca (AstraZeneca, London, UK) and GlaxoSmithKline (GlaxoSmithKline, Middlesex, UK), respectively, and were restored in dimethyl sulfoxide (DMSO), diluted in culture medium at 1:10,000 and stored at -20°C. Trastuzumab (Herceptin^®^, Hoffmann-La Roche Pharma, Basel, Switzerland) and cetuximab (Erbitux^®^, Merk-Serono, Darmstadt, Germany), provided by the Division of Pharmacy of the Catalan Institute of Oncology (Girona, Spain), were directly diluted in cell culture medium at 1:1,000 or 1:10,000 and were stored at 4°C. EGCG, EDTA, dithiotreitol, acetyl-CoA, malonyl-CoA, NADPH and 3,4,5-dimethylthiazol-2-yl-2,5-diphenyltetrazolium bromide (MTT) were purchased from Sigma (St. Louis, MO, USA). The primary antibody for FASN immunoblotting was a mouse IgG_1 _FASN monoclonal antibody from BD Biosciences Pharmingen (San Diego, CA, USA). Monoclonal anti-β-actin mouse antibody (clone AC-15) was from Sigma. Rabbit monoclonal antibodies against mTOR and phospo-mTOR^Ser2448 ^were from Cell Signaling Technology (Beverly, MD, USA). Rabbit polyclonal antibodies against PARP, ERK1/2 (extracellular signal-regulated kinase), phospo-ERK1/2 ^Thr202/Tyr204^, AKT, phospho-AKT^Ser473^, and mouse monoclonal p185^HER-2/neu ^were from Cell Signaling Technology. Peroxidase conjugated secondary antibody was from Calbiochem (San Diego, CA, USA). 1,3-*bis*((3,4,5-thihydroxybenzoil)oxy)naphthalene (G28UCM) was synthesized as previously described [13].

### Cell culture and cell lines

BT474 and AU565 breast carcinoma cells were obtained from the American Type Culture Collection (ATCC, Rockville, MD, USA). BT474 cells were cultured in DMEM-F12 (Gibco, Berlin, Germany) supplemented with 10% heat-inactivated fetal bovine serum (FBS, HyClone Laboratories, Logan, Utah, USA), 1% L-glutamine, 1% sodium pyruvate, 50 U/mL penicillin, and 50 μg/mL streptomycin (Gibco). AU565 cells were routinely grown in Dulbecco's Modified Eagle's Medium (DMEM, Gibco) supplemented as above. Trastuzumab-resistant cells (AU565TR) were developed [39,40] by exposing AU565 cells continuously to trastuzumab (0.4 μM for pool 0.4 and 2 μM for pool 2) for six months. Cells per plate were then pooled together and sensitivity to trastuzumab was determined by treating AU565 parental (AU565WT) and resistant (AU565TR) cells with 2 μM trastuzumab and performing trypan blue exclusion assay periodically during 10 days. Thus, cell pools which were resistant to trastuzumab were maintained in 2 μM trastuzumab, a concentration at which parental cells were not viable. To develop lapatinib-resistant cells (AU565LR), AU565 cells were treated for one month with an initial dose of 3.5 μM of lapatinib (IC_40 _of lapatinib in AU565WT cells), at which time the dose of lapatinib was increased up to 7 μM for five months. AU565LR cells were maintained in 7 μM lapatinib, a concentration at which AU565 parental cells were not viable.

### Growth inhibition and dose-response studies

Dose-response studies were done using standard colorimetric MTT reduction assay. Parental AU565 and trastuzumab- and lapatinib-resistant AU565 cells were plated out at a density of 7 × 10^3 ^cells/100 μL/well in 96-well microtitre plates. Following overnight cell adherence, the medium was removed and fresh medium along with the corresponding concentrations of FASN inhibitors (EGCG and G28UCM) or anti-HER agents (trastuzumab, cetuximab, erlotinib, gefitinib and lapatinib) were added to the cultures. For the drug-combination experiments a dose concentration of G28UCM (5 to 40 μM) and EGCG (20 to 150 μM) plus different fixed concentrations of trastuzumab, cetuximab, erlotinib, gefitinib and lapatinib, were added to the microtitre culture plates. The concentrations of the anti-HER2 agents were determined from dose-response experiments in AU565 cells (data not shown). Agents were not renewed during the entire period of cell exposure (48 h for erlotinib, gefitinib or lapatinib and 72 h for trastuzumab or cetuximab), and control cells without agents were cultured under the same conditions with comparable media changes. Following treatment, the media was replaced by drug-free medium (100 μL/well) containing MTT solution (10 μL, 5 mg/ml in PBS), and incubation was prolonged for 3 h at 37°C. After carefully removing the supernatants, the formazan crystals formed by metabolically viable cells were dissolved in DMSO (100 μL/well) and the absorbance was determined at 570 nm in a multi-well plate reader (Model Rosyf Anthos 2010, Anthos Labtec B.V., Heerhugowaard, Nederland). Using control optical density (OD) values (*C*), test OD values (*T*), and time zero OD values (*T_0_*), the compound concentration that caused 50% growth inhibition (IC_50 _value) was calculated from the equation, 100 × ((*T - T_0_*)/(*C - T_0_*)) = 50. The data presented are from three separate wells per assay and the assay was performed at least three times.

### Isobologram analysis of drug interactions

The interactions of G28UCM and EGCG with anti-HER drugs (trastuzumab, lapatinib, gefitinib, erlotinib and cetuximab) were evaluated by the isobologram method as we have previously published [41,42]. Briefly, the concentration of one agent producing a 30% inhibitory effect is plotted on the horizontal axis, and the concentration of another agent producing the same degree of effect is plotted on the vertical axis; a straight line joining these two points represents zero interaction (addition) between two agents. The experimental isoeffect points were the concentrations (expressed relative to the IC_30 _concentrations) of the two agents that when combined kill 30% of the cells. When the experimental isoeffect points fell below that line, the combination effect of the two drugs was considered to be supra-additive or synergistic, whereas antagonism occurs if the experimental isoeffect points lie above it. Within the designed assay range, a set of isoeffect points was generated because there were multiple FASN inhibitors and anti-target agent concentrations that achieved the same isoeffect. A quantitative index of these interactions was provided by the equation I_x _= (A/a) + (B/b), where, for this study, *a *and *b *represent the respective concentrations of FASN inhibitors (EGCG or G28UCM) and anti-HER agents (trastuzumab, cetuximab, erlotinib, gefitinib and lapatinib) required to produce a fixed level of inhibition (IC_30_) when administered alone, and *A *and *B *represent the concentrations required for the same effect when the drugs were administered in combination, and I_x _represents an index of drug interaction (interaction index). I_x _values of < 1 indicate synergy, a value of 1 represents addition, and values of > 1 indicate antagonism. For all estimations of I_x_, we used only isobolos where intercept data for both axes were available.

### Western blot analysis of tumour and cell lysates

Cells and animal tumour tissues were collected and lysed in ice-cold lysis buffer containing 1 mM EDTA, 150 mM NaCl, 100 μg/mL PMSF, 50 mM Tris-HCl (pH 7.5), protease and phosphatase inhibitor cocktails (Sigma). A sample was taken for measurement of protein content by Lowry-based BioRad assay (BioRad Laboratories, Hercules, CA, USA) and either used immediately or stored at -80°C. Total protein extracts were immunoblotted using 3% to 8% SDS-PAGE (FASN, p185^HER2/neu^, phospho-p185^HER2/neu^, mTOR and phospho-mTOR) or 4% to 12% SDS-PAGE (AKT, phospho-AKT, ERK1/2 and phospo-ERK1/2 and PARP), transferred to nitrocellulose membranes and blocked for 1 h in blocking buffer at room temperature (2.5% powdered-skim milk in PBS-T (10 mM Tris-HCL pH 8.0, 150 mM NaCl and 0.05% Tween-20)) to prevent non-specific antibody binding. Blots were incubated overnight at 4°C with the corresponding primary antibody diluted in blocking buffer. After washes in PBS-T (3 × 5 minutes), blots were incubated for 1 h with the corresponding secondary antibody and revealed, employing a commercial kit (West Pico chemiluminescent substrate). Blots were re-probed with an antibody for β-actin to control for protein loading and transfer.

### *In vivo *studies: human breast tumour xenograft experiments

Experiments were conducted in accordance with guidelines on animal care and use established by Biomedical Research Institute of Bellvitge (IDIBELL) Institutional Animal Care and Scientific Committee. The BT474 cell line was selected for the *in vivo *studies due to its high constitutive FASN and HER2 expression and its *in vivo *behavior, as we have previously reported [13]. A dose of G28UCM of 40 mg/Kg was chosen for efficacy experiments. Ten female mice were included in the control group and 14 in the G28UCM-treated group. Tumour xenografts were established by subcutaneous injection of 10 × 10^6 ^BT474 cells mixed in Matrigel (BD Bioscience, Bedford, MA, USA) into the flank. Tumours were allowed to increase up to a size of 150 to 250 mm^3^. Mice were treated by intraperitoneal injection daily with 40 mg/Kg of G28UCM or vehicle for 45 days. Mice were weighed once per week, tumours were measured daily with electronic calipers, and tumour volumes were calculated by the formula: (π/6 × (v1 × v2 × v2)), where v1 represents the largest tumour diameter, and v2 the smallest one. At the end of the experiment, animals were weighed and all mice were euthanized, and tumours, brain, lung, heart, liver, spleen, intestine and kidney tissues and serum were stored at -80°C.

### *In vivo *studies: animal toxicity experiments

Experiments were conducted in accordance with guidelines on animal care and use established by Biomedical Research Institute of Bellvitge (IDIBELL) Institutional Animal Care and Cientific Committee (AAALAC unit 1155). The study protocol has received ethical approval. Female athymic nude BALB/c mice (four to five weeks old, 23 to 25 g) were purchased from Harlan Laboratories (France), fed *ad libitum *with a standard rodent chow and housed in a light/dark 12 h/12 h cycle at 22°C in a pathogen-free facility for one week. Animals were randomized into four groups of six animals each: control, 5, 40 and 75 mg/Kg G28UCM-treated animals. Each group received daily a single intraperitoneal (i.p.) injection (0.5 mL) of G28UCM (5, 40 and 75 mg/Kg) or vehicle alone (DMSO), dissolved in RPMI 1640 medium. The body weight was registered daily for 45 days. On day 45 animals were sacrified and renal (urea and creatinin) hepatic (aspartate transaminase, alanine trasaminase and alkaline phosphatase) function markers, and hematological parameters (% neutrophils, % lymphocytes, % monocytes, % platelets, hemoglobine and % hematocrit) were determined in serum of control and G28UCM-treated animals.

### *Ex vivo *immunohistochemistry of FASN

Immunohistochemical staining for FASN was performed using a rabbit monoclonal antibody anti-FASN (Assay Designs, Ann Arbor, MI, USA). Briefly, paraffin-embedded tissue sections of control and G28UCM-treated xenografts were deparaffinized, rehydrated, and blocked with 2% hydrogen peroxide for endogenous peroxidase. Slides were washed with phosphate-buffered saline (PBS) and blocked with 20% horse serum (JRH Bioscience, Lexena, KS, USA). Slides were then incubated with anti-FASN antibody overnight at 4°C. After additional PBS washes, sections were sequentially incubated at room temperature for 45 minutes with biotin-labeled antirabbit IgG (Envision + R System Labelled Polymer-HRP anti-rabbit, Dako, Aachen, Germany). Slides were washed with PBS and incubated with diaminobenzidine (DAB, Sigma Chemical, St. Louis, MO). Finally, slides were counterstained with Hematoxylin-eosin, dehydrated, cleared and cover-slipped. FASN expression was categorized as negative (no or weak expression) or positive (strong expression). Appropriate positive and negative controls were included in each run of immunohistochemistry. All immunohistochemically stained slides were interpreted by a pathologist blinded to other data.

### Fluorescent *in situ *hibridation (FISH)

Cytospin slides of AU565 parental and resistant cells to trastuzumab or lapatinib were prepared. The HER2 FISH pharmDX™ Kit (Dako, Aachen, Germany) was used as directed by the manufacturer. Slides were heated in Pre-Treatment Solution for 10 minutes, and digested with ready-to-use pepsin at room temperature for 5 to 10 minutes. A ready-to-use FISH probe mix was hybridised onto slides. This probe mix consists of a mixture of Texas Red-labelled DNA probes covering a 218 kb region including the HER2 gene on chromosome 17 (CEN17), and a mixture of fluorescein-labelled peptide nucleic acid (PNA) probes targeted at the centromeric region of CEN17. The specific hybridisation to the two targets results in formation of a distinct red fluorescent signal at each HER2 gene locus and a distinct green fluorescent signal at each chromosome 17 centromere. After a stringent wash with the buffer the slides were mounted with fluorescent mounting medium containing DAPI and coverslipped. Twenty nuclei were assessed for HER2 and CEN17. The ratio of average HER2 to average CEN17 copy number was calculated. Gene amplification was defined when the FISH ratio HER2 signal/CEN17 signal was > 2.

### Statistical analysis

Results were analysed by Student's *t*-test or by one-way ANOVA using a Tukey test as a post-test. Statistical significant levels were *P *< 0.05 (denoted as *) and *P *< 0.005 (denoted as**). All data are means ± standard deviation (SD) or ± standard error (SE). All observations were confirmed by at least three independent experiments.

## Results

### Efficacy of G28UCM against breast carcinoma xenografts

**Figure 1:**
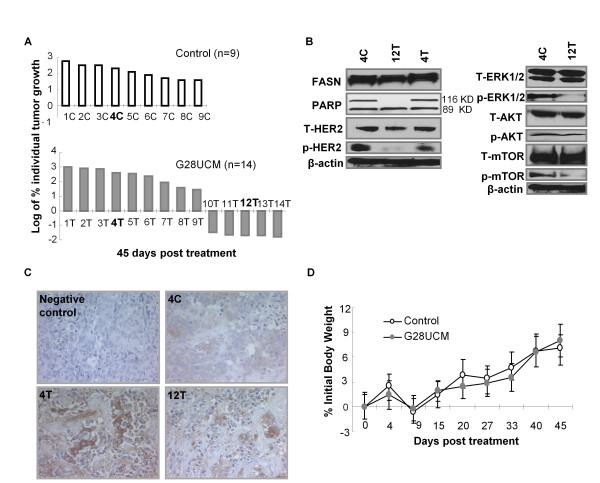
**G28UCM inhibits the growth of BT474 xenografts and do not induce weight loss *in vivo***. **A**. Daily i.p. 40 mg/Kg G28UCM-treatment decreased tumour volume in a BT474 breast cancer xenograft (*n *= 14, grey bars) compared to vehicle control (*n *= 9, white bars). Five G28UCM-treated animals exhibited no identifiable residual tumour at the end of the experiment (10T, 11T, 12T, 13T and 14T, grey bars). Data are expressed as logarithm of percentage of individual tumour growth at day 45 respect to day 0. **B**. G28UCM-treated tumours showed apoptosis and inactivation of HER2, ERK1/2 and mTOR signalling pathways, without affecting FASN protein expression levels. This figure only shows a representative animal of each experimental group (4C -control-, 4T -treated no-responsive- and 12T -responsive-). All tumours were lysed and equal amounts of protein were subjected to Western blot analyses with anti-PARP, anti-FASN, anti-HER2, anti-AKT, anti-ERK1/2 and anti-mTOR antibodies. Activation of the protein under study was analysed by assessing the phosphorylation status using the corresponding phospho-specific antibody. Blots were reprobed for β-actin as loading control. Gels shown are representative of those obtained from two independent experiments. **C**. FASN expression level does not change between control and G28UCM-treated animals. Representative immunohistochemical staining for FASN protein of xenograft tumour of untreated (4C) and G28UCM-treated non-responding (4T) and responding (12T) group. **D**. G28UCM treatment does not induce weight loss. The body weight of each mouse was measured before and weekly after treatment with G28UCM (40 mg/Kg/day for 45 days) or vehicle control. Data are expressed as percentage of initial body weight and represent mean values ± SE for each experimental group.

Blocking FASN activity causes cytotoxicity in human cancer cells overexpressing FASN [2-13]. The proposed oncogenic properties of FASN seem to be the result of an increased activation of HER2 and its downstream related signaling pathway proteins [4,5,8,13-20]. Because the *in vitro *studies were carried out for short-term periods, we further evaluated *in vivo *the long-term effect of G28UCM, a novel pharmacological inhibitor of FASN. BT474 human FASN+ and HER2+ breast carcinoma xenografts served as the tumour target for the *in vivo *studies. In all control animals, BT474 xenografts grew in size, reaching volumes at day 45 which were from 50% to 600% of the volumes at day 0 (first day of G28UCM treatment). The median size of the tumours when the experiments started was 127.4 ± 25.1 mm^3^. In the experimental animals, we observed two clear groups: in five cases, the xenografts experimented tumour volume reductions ranging from -20% to -90% (10T, 11T, 12T, 13T and 14T, Figure 1A), while in nine cases tumour growth was observed.

To analyse the activation of HER2 and its downstream related phosphoinositide-3 kinase/protein kinase B (PI3K/AKT) and mitogen-activated protein kinase/extracellular signal-regulated kinase (MAPK/ERK1/2) signalling cascades or to the mammalian target of rapamycin protein (mTOR) signalling pathway, we performed Western blotting and immunohistochemical analysis of each individual animal tumour. Apoptosis and induction of caspase activity were checked with cleavage of poly-ADP-ribose polymerase (PARP) in Western blotting analysis. Apoptosis was not detected in the tumours of control (C) and treated (T) animals with non-responding tumours. In contrast, in the tumours of G28UCM- responding animals, there was an increase in the levels of 89 kDa PARP product. Figure 1B shows the results of some representative tumours of each experimental group. We next examined the effects of G28UCM on HER2 and its related downstream proteins AKT, ERK1/2 and mTOR. Tumours that showed a response to G28UCM had a marked decrease in phosphorylated HER2, ERK1/2 and mTOR proteins and, to a lesser extent in phosphorylated AKT, without detectable changes in the total levels of the corresponding proteins. Figure 1B shows a representative result of each experimental group.

We also analysed FASN protein expression levels of each individual animal tumour. Results in Figure 1B depict FASN levels from one representative animal of the control group (4C, Figure 1A) and two G28UCM-treated animals (4T and 12T, Figure 1A). No significant changes in FASN protein levels were observed in any of the samples, as assessed both by Western blotting (Figure 1B) and either by immunohistochemical staining (Figure 1C). With respect to *ex vivo *FASN enzymatic activity (see Additional file 1), however, the experimental tumours that had a response to G28UCM (11T and 12T, Figure 1A) showed a decrease of 30.5 ± 15% compared with the control 4C tumour (see Additional file 2).

### Toxicity studies

Previous first-generations of FASN inhibitors have been limited by inducing severe body weight loss, which is thought to be related to a parallel stimulation of fatty acid oxidation by these inhibitors [4,6,24-28]. To address this problem, G28UCM were designed to inhibit FASN activity without parallel stimulation of *in vitro *fatty acid oxidation [13]. In this study, animals treated for 45 days with G28UCM were weighed daily to evaluate *in vivo *body weight effect of the novel FASN inhibitor. With respect to control animals, we identified no significant changes on food and fluid intake or body weight after daily treatment with 40 mg/Kg of G28UCM for 45 days. The average weight of the animals at the beginning of the study was 19.8 ± 1.7 g. At the conclusion of the study, control animals increased their weight by 7.15 ± 0.8% of pre-treatment weight, compared with 8.04 ± 1.6% for the G28UCM**-**treated animals (Figure 1D) which was not statistically significant.

Hepatic and renal function serum markers (aspartate transaminase, alanine transaminase, alkaline phosphatase, creatinin and urea) showed no significant alteration between control and experimental animals treated with G28UCM at daily doses of 5, 25 or 40 mg/Kg. Animals treated at doses of 75 mg/Kg, however, showed differences compared with control in their blood counts, in particular, increased neutrophils and platelet cells and decreased monocytes and lymphocytes (see Additional file 3 for hepatic, renal and hematological function serum markers of G28UCM-treated animals). Histological studies (Hematoxylin-Eosin, Masson's Trichrome, Sudan Black B and Picrosirius Red stains) of liver, heart, kidney, lung and brain showed no tissue structural abnormalities in G28UCM-treated animals when compared with control animals (data not shown).

### *In vitro *cell growth interactions between G28UCM and anti-HER drugs

To determine how best to use G28UCM either as a single agent or in combination with anti-HER drugs, we conducted a series of *in vitro *studies to evaluate the inhibitory effects of G28UCM in combination with trastuzumab, cetuximab, erlotinib, gefitinib and lapatinib in a pre-clinical model of HER2-overexpressing breast cancer cells. The combined effect was analysed by the isobole method, using a series of isobologram transformations of multiple dose-response curves at an effect level of 30% (IC_30_), a type of analysis that we have used previously [41,42]. Results in Table 1 show the median interaction index (Ix) of combinations between G28UCM with trastuzumab, cetuximab, erlotinib, gefitinib and lapatinib. Simultaneous treatment of AU565 cells with G28UCM and either trastuzumab, lapatinib, gefitinib or erlotinib resulted in a strong synergistic interaction (I_x _= 0.519 ± 0.178, I_x _= 0.796 ± 0.144, 0.882, I_x _= 0.832 ± 0.161 and I_x _= 0.735 ± 0.092, respectively). The combination of G28UCM plus cetuximab indicated a marked antagonistic interaction (I_x _= 1.913 ± 0.243). Under the same schedule, EGCG showed an additive interaction with trastuzumab (I_x _= 1.123 ± 0.458) and antagonistic interactions with lapatinib, gefitinib and erlotinib and cetuximab (I_x _= 1.875 ± 0.691, I_x _= 1.829 ± 0.672, I_x _= 1.393 ± 0.229, I_x _= 2.156 ± 0.215, respectively). Together, these data show that co-exposure of the FASN inhibitor G28UCM with drugs that exhibit anti-HER2 activity (but not with the specific anti-HER1 compound, cetuximab) is more active than either of the drugs used alone.

**Table 1:** Synergy analysis of the interaction between G28UCM and anti-HER drugs in AU565 cells

*plus*	Anti-HER2		Anti-HER1		
	
	trastuzumab	lapatinib	gefitinib	erlotinib	cetuximab
G28UCM	synergism 0.5 ± 0.2**	synergism 0.8 ± 0.1**	synergism 0.8 ± 0.2*	synergism 0.7 ± 0.1**	antagonism 1.9 ± 0.2**
EGCG	additivism 1.1 ± 0.5	antagonism 1.9 ± 0.7*	antagonism 1.8 ± 0.7*	antagonism 1.4 ± 0.2*	antagonism 2.2 ± 0.2**

The interaction index (I_x_) for the two-drug effect in AU565 cells was calculated using isobologram analysis. The I_x _parameter indicate whether the doses of the two drugs required to produce a given degree of cytotoxicity are greater than (I_x _> 1 or antagonism) equal to (I_x _= 1 or additivism) or less than (I_x _< 1 or synergism) the doses that would be required if the effect of two agents were strictly additive. I_x _values for the two drug treatment were obtained from triplicate studies. * (*P *< 0.05) and ** (*P *< 0.005) indicate the level of statistical significance of the I_x _compared with an I_x _of 1.0.

### Molecular interactions between G28UCM and anti-HER drugs

**Figure 2:**
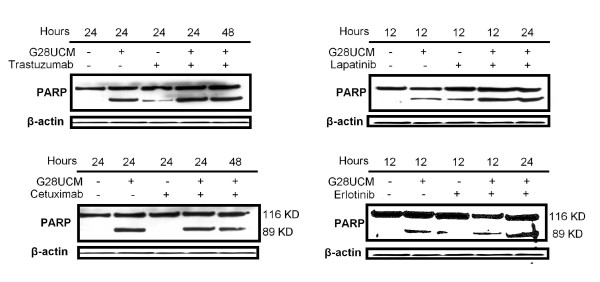
**G28UCM plus trastuzumab, lapatinib and erlotinib induced apoptosis in AU565 breast cancer cells**. Induction of caspase activity was confirmed by PARP cleavage. AU565 cells were treated with G28UCM (30 μM) plus trastuzumab (1 μM), lapatinib (5 μM), erlotinib (8 μM) or cetuximab (15 μg/ml) for 12 and 24 h (G28UCM *plus *lapatinib or erlotinib), and 24 and 48 h (G28UCM *plus *trastuzumab or cetuximab), and equal amounts of lysates were immunoblotted with anti-PARP antibody which identified the 116 KDa (intact PARP) and the 89 KDa (cleavage product) bands. Blots were reprobed for β-actin as loading control. Gels shown are representative of those obtained from two or three independent experiments.

**Figure 3:**
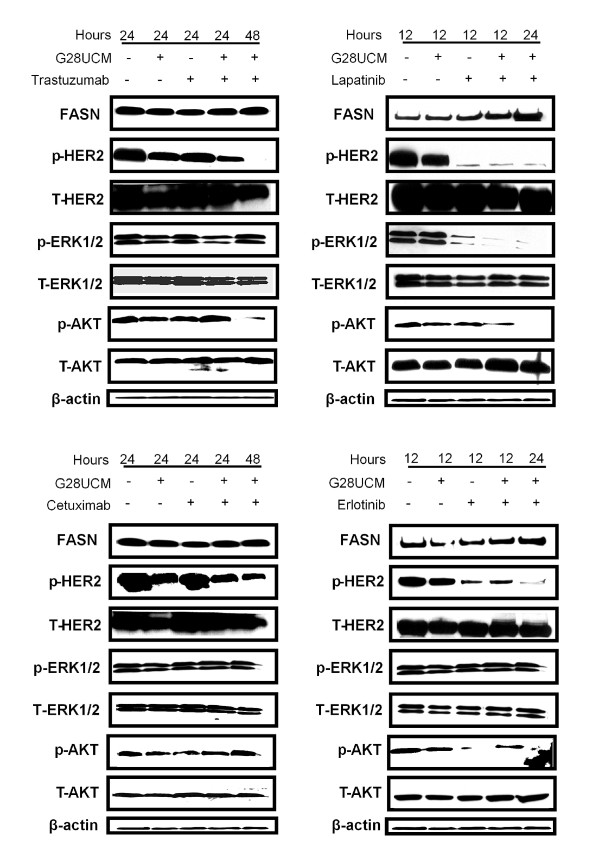
**G28UCM plus trastuzumab, lapatinib and erlotinib blocked the activation of HER2, AKT or ERK1/2 proteins**. AU565 cells were treated with G28UCM (30 μM) plus trastuzumab (1 μM), lapatinib (5 μM), erlotinib (8 μM) or cetuximab (15 μg/ml) for 12, 24, and 48 h, and equal amounts of lysates were immunoblotted with anti-FASN, anti-HER2, anti- AKT, and anti-ERK1/2 antibodies. Activation of the protein under study was analysed by assessing the phosphorylation status using the corresponding phospho-specific antibody. Blots were reprobed for β-actin as loading control. Gels shown are representative of those obtained from two or three independent experiments.

To determine whether the molecular causes of the synergistic interactions between G28UCM and trastuzumab, lapatinib, cetuximab and erlotinib were triggered by changes in the phosphorylated forms of HER2 and its downstream signaling proteins, we analysed changes in apoptosis and HER2, AKT and ERK1/2 protein phosphorylated forms. First, we studied the cell death mechanism. Apoptosis and induction of caspase activity were checked by Western blotting analysis showing cleavage of PARP. The experiments were done at a concentration equal to the cytotoxicity IC_50 _value of G28UCM and anti-HER drugs (trastuzumab, lapatinib, cetuximab and erlotinib) in AU565 cells. Co-treatment of AU565 cells with G28UCM (30 μM) plus trastuzumab (1 μM) during 24 h induced a marked increase in the levels of the PARP cleavage product (89 kDa band) compared to 24 h single agent (G28UCM or trastuzumab) treatment (Figure 2). The apoptotic effect of the combined regimes was validated by flow cytometry using the Annexin V-Alexa Fluor 488 staining (data not shown). Similar results in PARP cleavage were obtained when AU565 cells were co-treated with G28UCM (30 μM) plus lapatinib (5 μM) during 12 hours or plus erlotinib (8 μM) during 24 hours (Figure 2). Therefore, we sought to compare the effects of combined treatments versus single drug treatments on HER2, AKT, and ERK1/2 activation. The phosphorylated form of HER2 (p-HER2) was noticeably decreased after 24 h exposure to G28UCM plus trastuzumab, and p-AKT protein decreased after 48 h of co-treatment with G28UCM and trastuzumab (Figure 3). Co-incubation of cells with G28UCM and lapatinib was significantly correlated with a decreased level of the phosphorylated form of HER2 (pHER2) and p-ERK1/2, which occurred as soon as 12 h after treatment compared to 12 h cell treatment with either G28UCM or lapatinib alone (Figure 3). Co-exposure of G28UCM plus erlotinib induced a decrease of p-HER2 and p-AKT after 24 hours (Figure 3). During all time-course co-treatment experiments no significant change either in the total level of the corresponding proteins (HER2, ERK1/2 and AKT) or in FASN levels was detected (Figure 3).

As we expected, under the same culture conditions, co-treatment of AU565 cells with G28UCM plus cetuximab (15 μg/mL) did not induce apoptosis (Figure 2) and did not block HER2 phosphorylation or its downstream related signal transduction pathways ERK1/2 and PI3K/AKT (Figure 3).

### Effect of G28UCM on cells resistant to trastuzumab or lapatinib

**Figure 4:**
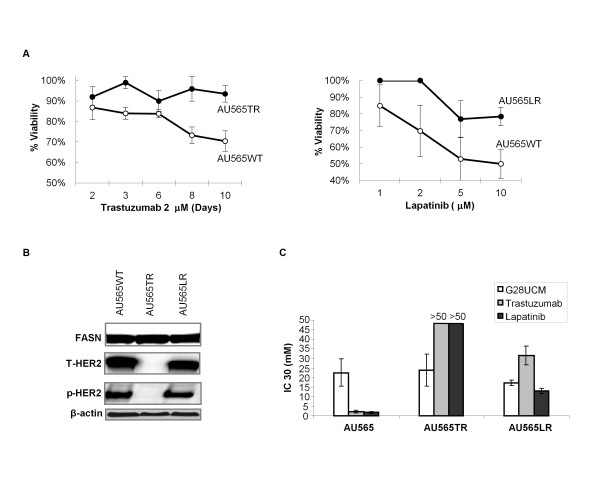
**G28UCM shows cytotoxic activity in developed HER2 + and FASN + trastuzumab and lapatinib-resistant cells**. **A**. Development of trastuzumab- and lapatinib-resistant AU565-derived cells. AU565 parental cells (with ○) and AU565 cells cultured for six months in 2 μM of trastuzumab (AU565TR, with ●) were both treated with 2 μM of trastuzumab for 2, 3, 6, 8 and 10 days. Cells were trypsinized and counted by trypan blue exclusion. Results are shown as the percentages of viable cells compared with untreated control cultures for each cell line and period-time. All experiments were repeated at least two times. AU565 parental cells (with ○) and AU565 cells cultured for one month in 3.5 μM and the next five months in 7 μM of lapatinib (AU565LR, with ●) were both treated in with different concentrations of lapatinib (1 to 10 μM) for 48 h. Circles represent the percentage of surviving cells after 48 h in lapatinib treatment, which was determined using an MTT assay. Results are expressed as percentage of surviving cells from three independent experiments performed in triplicate (mean ± SD). **B**. FASN and HER2 expression levels in parental and resistant cells. AU565 parental and resistant cells (AU565TR and AU565LR) were lysed for protein and immunoblotted for FASN, T-HER2, p-HER2. Blots were reprobed for β-actin as loading control. Gels shown are representative of those obtained from three independent experiments. **C**. Cytotoxicity in AU565 parental and resistant cells (AU565TR and AU565LR) following G28UCM treatment. AU565 parental and resistant cells were treated along with different concentrations of G28UCM (1 to 40 μM), trastuzumab (1 to 50 μM) or lapatinib (1 to 50 μM). Results represent IC30 values of G28UCM, trastuzumab and lapatinib in AU565 parental and resistant cells (AU565TR and AU565LR), which was determined using an MTT assay. Results are expressed as mean IC30 values ± SD from three independent experiments performed in triplicate.

The vast majority of HER2 positive advanced breast cancer patients develop resistance to trastuzumab based therapies within the first year of treatment. Consequently, identification of novel agents that inhibit the growth of trastuzumab-resistant cells/tumours is critical to improving the survival of metastatic HER2+ breast cancer. For this purpose, we extended our study to examine the anti-cancer effect of G28UCM on HER2+ breast cancer cells (AU565) that were continuously exposed in culture medium supplemented with trastuzumab (AU565TR) or lapatinib (AU565LR) over a period of at least six months. Trastuzumab resistant (AU565TR) or lapatinib resistant (AU565LR) cells were developed in our laboratory as described in the Materials and methods section. Sensitivity to trastuzumab was determined by treating AU565 parental and resistant cells to 2 μM trastuzumab and performing trypan blue exclusion assay periodically during 10 days (Figure 4A, left). A dose of 2 μM trastuzumab caused a significant cell death in AU565 cells (70.2 ± 5%), but the majority of AU565TR cells remained viable (94.6 ± 7%). Lapatinib resistance was confirmed by an MTT colorimetric assay (Figure 4A, right).

To eliminate the possibility that we have selected a population of resistant cells that do not possess HER2 gene amplification, we examined HER2 gene amplification by fluorescence *in situ *hybridisation using a method that determines oncogene copy number corrected to the number of copies of chromosome 17 (CEP17). The ratio of the average HER2 gene copy number to the average CEP17 gene copy number in AU565TR was 3.9, 4.9 in AU565WT, and 4.4 in AU565LR respectively, demonstrating that both trastuzumab and lapatinib resistant cells possess HER2 amplification similar as parental cells (Table 2).

**Table 2:** FISH* analysis of HER2 gene copy number in AU565WT, AU565TR and AU565LR cells

CELL LINES	AU565WT	AU565TR	AU565LR
Ratio of average HER2 to average CEN17 gene copy number^†^	4.9	3.9	4.4

AU565WT, AU565 Wild Type; AU565TR, AU565 cells resistant to trastuzumab; AU565LR, AU565 cells resistant to lapatinib;

* FISH, fluorescence *in situ *hybridisation; ^† ^HER2/CEN17 > 2 indicates HER2 gene amplification.

Additionally, we performed immunoblotting experiments to determine HER2, pospho-HER2 (pHER2) and FASN protein levels in AU565TR and AU565LR cells. HER2 and pHER2 were down-regulated in AU565TR cells (Figure 4B). In AU565LR cells, protein levels of HER2 and pHER2 did not change compared with AU565WT cells and FASN levels were similar in the three cell lines (Figure 4B). To analyse the sensitivity of the resistant cells to G28UCM, we determined the growth inhibition effect of this compound by an MTT colorimetric assay, using trastuzumab and lapatinib as reference compounds. As expected, trastuzumab and lapatinib had either no effect or a weak effect on growth inhibition of trastuzumab- and lapatinib-resistant cells, respectively (Figure 4C). For instance, while the IC_30 _value of trastuzumab in AU565WT was 2 μM, AU565TR cells were insensitive to trastuzumab at the concentrations analysed (up to 50 μM of trastuzumab). The IC_30 _value of lapatinib was increased from 1.6 μM in AU565WT to 14 μM in AU565LR (Figure 4C). Trastuzumab concentration necessary to achieve IC_30 _value had to be increased about 16-fold in AU565LR (IC_30 _= 31.5 ± 4.9 μM) compared to AU565WT (IC_30 _= 2 ± 0.7 μM), and lapatinib had no cytotoxic activity in AU565TR cells using doses up to 50 μM (Figure 4C). Interestingly, G28UCM showed similar cytotoxic activity in parental (IC_30 _= 22 ± 7 μM), trastuzumab- (IC_30 _= 24 ± 8 μM) and lapatinib-resistant cells (IC_30 _= 17 ± 2 μM). Taken together, these data suggest that inhibiting FASN activity may be a new therapeutic strategy in breast carcinomas with acquired resistance to anti-HER2 therapies.

## Discussion

Treatment with G28UCM was associated with xenograft volume reductions from 20% to 90%, in 5 of 14 animals. The responding tumour tissues showed changes in apoptosis and in HER2-related signalling pathways. They showed an increase in the levels of 89 kDa PARP product, and the phosphorylated forms of HER2 (pHER2), ERK1/2 (pERK1/2) and mTOR (pmTOR) were almost abolished. These samples showed a decline in FASN enzymatic activity, but not total FASN levels. It is not clear why a substantial number of xenografts did not respond to G28UCM. The degree of interindividual variability in the response to G28UCM might be related to bioavailability, clonal variation or experimental design. Concerning bioavailability, G28UCM reached the target tissue in the responding xenografts, since the *in vivo *FASN inhibition was of 30% (see SD), which is similar to the reported intra-tumour 40% inhibition of FASN activity 12 hours after intraperitoneal injection of other FASN inhibitors [43]. Non-responding tumours, in contrast, had no detectable changes in apoptosis or pHER2, pERK or pmTOR expression after treatment with G28UCM. The observed inhibition was able to elicit clear molecular responses in at least one-third of the treated animals. Clonal variability of BT474 cells cannot be excluded. In fact, Sheridan *et al*. described that 80% of BT474 cells in culture expressed CD24, while 20% did not [44]. The relevance of CD24, a cell adhesion molecule, in our system is not clear. Furthermore, for the sake of therapeutic significance, our experimental design consisted of administration of G28UCM after the xenografts had reached a size of 100 to 150 mm^3^. It is possible that treating smaller tumours or administering G28UCM at the same time as the human cells might translate into a less variable result. Future experiments will need to explore in detail the pharmacokinetics and pharmacodynamics of the compound in this model, develop alternative animal and xenograft models, as well as alternative routes of administration of the compound. These *in vivo *data seem to confirm that the oncogenic properties of FASN could be associated with an increased phosphorylation of HER2, and its related PI3K/AKT, MAPK/ERK1/2, and mTOR signaling cascades [4,5,8,13-20]. In this report we did not address the issue of the extent to which the effects of G28UCM are mediated by inhibition of FASN alone or by off-target effects, since we have reported previously on this relationship [13]. Future experiments, however, will address the specificity of G28UCM against FASN. This is particularly important since the parent molecule of G28UCM has been reported to have an array of biological activities, including the inhibition of gelatinase-B (MMP-2), NO synthase or aromatase enzymatic activities [45-47].

An important part of our *in vivo *results concerns the toxicity of G28UCM. We performed a long-term weight evaluation, and no significant effect on food and fluid intake or body weight was identified after daily treatment with 40 mg/Kg of G28UCM for 45 days. In addition, hepatic and renal function serum markers and histological studies of liver, heart, kidney, lung and brain showed no significant alterations between control and animals treated during 45 days with daily G28UCM. We suggest that the chemical structure of G28UCM may be more specific of the lipogenic pathway than cerulenin or its derivatives, which stimulate CPT-1 and accelerate fatty acid β-oxidation, which has been related to the severe decrease of food intake and induction of weight loss in rodents [24-28].

We found that the simultaneous treatment of FASN+/HER2+ breast cancer cells with G28UCM plus trastuzumab or lapatinib (which involve predominantly HER2), resulted in a strong synergistic interaction, and that this was also observed with gefitinib or erlotinib (inhibitors of HER1 but also HER2 tyrosine kinase activities) [48,49]. In contrast, the combination of G28UCM with the monoclonal antibody cetuximab (which is HER1-specific) resulted in an antagonistic effect. Taken together, these results support that the interactions between FASN and HER proteins are restricted to HER2 and do not involve the HER1 receptor. On the other hand, EGCG showed only an additive interaction with trastuzumab and an antagonistic interaction with lapatinib, gefitinib, erlotinib and cetuximab, which may be in part related to the lower cytotoxic activity of EGCG by itself. We also addressed the molecular interactions of G28UCM, analysing FASN protein levels, apoptosis, and the phosphorylated forms of HER2, AKT and ERK1/2 proteins after G28UCM combined with trastuzumab, erlotinib, gefitinib or lapatinib treatment. Trastuzumab and HER tyrosine kinase inhibitors (lapatinib, gefitinib and erlotinib) displayed molecular synergistic interaction with G28UCM. This synergistic effect was accompanied by increased apoptosis and seemed to be mediated by abrogation of the activation of HER2, AKT and ERK1/2 when the drugs are combined. It is important that the synergistic molecular effects observed with G28UCM in combination with trastuzumab, erlotinib, gefitinib or lapatinib followed the same pattern than the cellular effects. These *in vitro *cellular and molecular synergistic results support the *in vivo *evaluation of these agents in a combination regimen.

Finally, we used stable cell lines derived from the AU565 cells that were resistant to either trastuzumab (AU565TR) or lapatinib (AU565LR) to test the anticancer properties of G28UCM. In these cells, in which the cytotoxicity of trastuzumab and lapatinib were almost lost, we observed that the cytotoxic activity of G28UCM in the resistant cells and in the parental cells was similar. The activity of G28UCM in this model of resistance to anti-HER2 treatments is consistent with a previous report that observed that trastuzumab-resistant breast cancer cells were sensitive to EGCG [50]. Furthermore, our results also show that, even after long-term exposure to trastuzumab and lapatinib, resistant cells continued to overexpress FASN.

## Conclusions

In summary, our findings provide a rationale for the pre-clinical development of G28UCM either alone or in combination with anti-HER agents (trastuzumab, lapatinib, erlotinib, gefitinib or cetuximab) in HER2-overexpressing breast cancer. In addition, we report the effect of G28UCM on breast cancer cells resistant to trastuzumab or lapatinib. Our data support the study of G28UCM as a potential therapeutic agent, either alone or in combination, against *in vivo *HER2+ tumours that have progressed on trastuzumab and lapatinib. Future studies will focus on testing the *in vivo *activity of G28UCM in mice bearing trastuzumab and lapatinib resistant xenografts.

## Abbreviations

AKT: protein kinase B; ANOVA: analysis of variance; CPT-1: carnitine palmitoyltransferase-1; EGCG: (-)-epigallocatechin-3-gallate; EGF: epidermal growth factor; FASN: fatty acid synthase; FISH: fluorescent *in situ *hibridation; HER2: human epidermal growth factor receptor 2; MAPK/ERK1/2: mitogen-activated protein kinase/extracellular signal-regulated kinase; i.p.: intraperitoneal; mTOR: mammalian target of rapamycin protein: MTT: 3-(4,5-dimethyl-2-thiazolyl)-2,5-diphenyl-2*H*-tetrazolium bromide; PARP: poly-ADPribose polymerase; PBS: phosphate-buffered saline; PI3K: phosphatidylinositol-3-OH-kinase; SREBP-1c: sterol regulatory element-binding.

## Competing interests

None of the authors have any conflict of interest that can affect the impartiality of the research reported.

## Authors' contributions

TP conceived of the study, helped in the molecular and cell biology studies, participated in the study design and coordination, and drafted the manuscript. HA and SC carried out the *in vivo *efficacy and toxicity studies. GO carried out the development of the resistant cells, FISH experiments, and *in vitro *and *ex vivo *FASN enzymatic activity assays. CT carried out the synthesis of G28UCM. SO-G, BB and MLL-R participated in the design and development of G28UCM and reviewed the manuscript. AU and RC conceived of the study and participated in the study design and coordination, and helped to draft the manuscript. All authors read and approved the final manuscript.

## Supplementary Material

Additional file 1 **Additional Material and methods on e*x vivo *FASN enzymatic activity assay**. Click here for file

Additional file 2 **Figure**. FASN activity decrease in G28UCM-treated responsive animal. Twelve hours after the last i.p. G28UCM injection, tumour tissues from a representative animal of control (4C) and G28UCM-treated responding group (12T) were minced and homogenized in ice-cold lysis buffer and FASN activity was assayed in particle-free supernatants by recording spectrophotometrically at 37°C the decrease of A340 nm due to oxidation of NADPH after the addition of malonyl-CoA as described in the Materials and methods section. Data are mean ± SD from two separate experiments. Click here for file

Additional file 3 **Table**. Hepatic, renal and hematological function serum markers of G28UCM-treated animals. Click here for file
