# Supplementary material for: A novel inhibitor of fatty acid synthase shows activity against HER2+ breast cancer xenografts and is active in anti-HER2 drug-resistant cell lines
Source: Breast Cancer Res. 2011 Dec 16;13(6):R131. doi: 10.1186/bcr3077 (PMC3326573; doi:10.1186/bcr3077)
Supplement: Additional file 1 — Additional Material and methods on ex vivo FASN enzymatic activity assay. [file bcr3077-S1.DOC]

**ADDITIONAL FILE 1**

**Additional Materials and Methods**

***Ex vivo* fatty acid synthase activity assay**

FASN activity assay was done 12 hours after the last intraperitoneal (i.p.) injection. Tumor tissues were minced and homogenized in ice-cold lysis buffer (300 μL: 1 mM EDTA, 150 mM NaCl, 100 μg/mL PMSF, 50 mM Tris-HCl, pH 7.5) using the TissueRuptor. Then, tissues were sonicated during 30 minutes at 4ºC (PSelecta ultrasons) and centrifuged for 15 minutes at 4ºC to obtain supernatants particle-free. A supernatant sample was taken to measure protein content by the Lowry-based BioRad assay (BioRad). FASN activity assay was done as we previously described [8-9, 13]. Briefly, one-hundred and twenty micrograms of substrate were pre-incubated during 15 minutes at 37ºC in 0.2 M of potassium phosphate buffer pH 7.0, for temperature equilibration. The sample was then added to the reaction mixture: 200 mM potassium phosphate buffer pH 7.0, 1 mM EDTA, 1 mM dithiothreitol, 30 μM acetyl-CoA and 0.24 mM NADPH in 0.3 mL reaction volume were monitored at 340 nm for 3 min to measure background NADPH oxidation (Lambda Bio 20, Perkin Elmer, EUA; using UV Kinlab 2.80.02 software). After the addition of 50 μM of malonyl-CoA, the reaction was assayed for an additional 10 min to determine FASN-dependent oxidation of NADPH. Rates were corrected for the background rate of NADPH oxidation in the presence of acetil-CoA. FASN activity was expressed in nmol NADPH oxidized x min^-1^ x mg protein^-1^.

.
