# Supplementary figures and images for: A novel inhibitor of fatty acid synthase shows activity against HER2+ breast cancer xenografts and is active in anti-HER2 drug-resistant cell lines
Source: Breast Cancer Res. 2011 Dec 16;13(6):R131. doi: 10.1186/bcr3077 (PMC3326573; doi:10.1186/bcr3077)

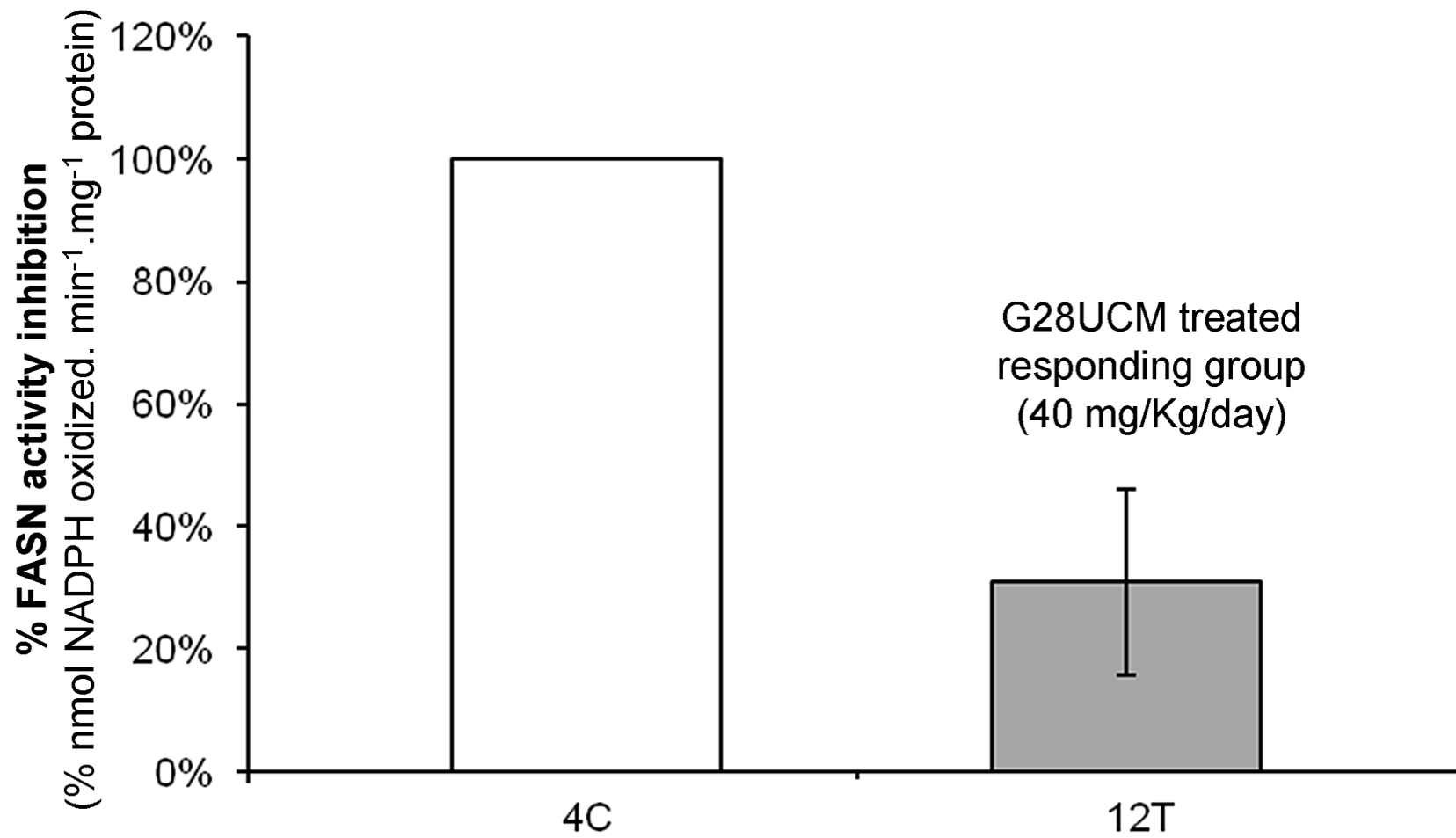

Supplement: Additional file 2 — Figure. FASN activity decrease in G28UCM-treated responsive animal. Twelve hours after the last i.p. G28UCM injection, tumour tissues from a representative animal of control (4C) and G28UCM-treated responding group (12T) were minced and homogenized in ice-cold lysis buffer and FASN activity was assayed in particle-free supernatants by recording spectrophotometrically at 37°C the decrease of A340 nm due to oxidation of NADPH after the addition of malonyl-CoA as described in the Materials and methods section. Data are mean ± SD from two separate experiments. [file bcr3077-S2.PDF]
