# Supplementary material for: A novel inhibitor of fatty acid synthase shows activity against HER2+ breast cancer xenografts and is active in anti-HER2 drug-resistant cell lines
Source: Breast Cancer Res. 2011 Dec 16;13(6):R131. doi: 10.1186/bcr3077 (PMC3326573; doi:10.1186/bcr3077)
Supplement: Additional file 3 — Table. Hepatic, renal and hematological function serum markers of G28UCM-treated animals. [file bcr3077-S3.DOC]

**ADDITIONAL FILE 2**

**Additional Table. Hepatic, renal and hematological function serum markers of G28UCM-treated animals* ^*^BUN: urea; ^†^ALT: Alanine transaminase; ^‡^ALP: Alkaline phospatase; ^§^AST: Aspartate transaminase; ^**^ (p<0.05) indicate the level of statistical significance compared to vehicle control.**

|  | **Renal Function**  **(mg/dl)** | | **Hepatic Function**  **(U/L)** | | | **Haematological Parameters (%)** | | | | | |
| --- | --- | --- | --- | --- | --- | --- | --- | --- | --- | --- | --- |
|  | **BUN^*^** | **Creatinin** | **ALT^†^** | **ALP^‡^** | **AST^§^** | **Neutrophils** | **Lymphocytes** | **Monocytes** | **Platelet cells** | **Hematocrit** | **Hemoglobin (g/dl)** |
| **Vehicle**  **Control** | 40.6 + 6.4 | 0.4 + 0.1 | 162.7 + 22.5 | 109 + 26.8 | 642.3 + 406.6 | 25.6 + 7.5 | 60 + 6.2 | 2.2 + 0.6 | 660.2 + 295.1 | 49 + 3.6 | 14.8 + 0.5 |
| **G28UCM**  **5 mg/Kg** | 40.9 + 7.2 | 0.4 + 0.1 | 110.3 + 98.2 | 140.2 + 20.8 | 666.5 + 583.3 | 16.5 + 2.9 | 69.5 + 12.5 | 0.9 + 0.3 | 1045.3 + 186.2 | 46.3 + 2.7 | 14.6 + 1 |
| **G28UCM**  **40 mg/Kg** | 42.8 + 6.8 | 0.4 + 0.1 | 107.2 + 71 | 86.9 + 16.3 | 429.3 + 145.5 | 27 + 13.1 | 60 + 9.5 | 1.5 + 0.6 | 1028 + 186.2 | 45.5 + 4.5 | 13.4 + 1.5 |
| **G28UCM**  **75 mg/Kg** | 35.2 + 5.4 | 0.3 + 0.1 | 190.6 + 93.7 | 59.7 + 29.1 | 536.3 + 262.5 | 41.6 + 10.1 ****** | 41.3 + 7.7  ****** | 0.8 + 0.2  ****** | 1279.8 + 235.2  ****** | 45.2 + 1.3 | 13.5 + 0.5 |
